# Enhancing quality of life in epilepsy with a digital intervention (*emyna*): Results of the ELAINE randomized controlled trial

**DOI:** 10.1002/epi4.13014

**Published:** 2024-08-21

**Authors:** Björn Meyer, Linda T. Betz, Katja Brückner, Martin Holtkamp

**Affiliations:** ^1^ Research & Development Department GAIA Group Hamburg Germany; ^2^ Department of Neurology and Epileptology, Epilepsy Center Hamburg Evangelical Hospital Alsterdorf Hamburg Germany; ^3^ Department of Neurology, Epilepsy‐Center Berlin‐Brandenburg Charité‐Universitätsmedizin Berlin Berlin Germany

**Keywords:** CBT‐based online program, cognitive behavioral therapy, digital therapeutic, epilepsy, internet intervention, lifestyle change

## Abstract

**Objective:**

Despite the availability of pharmacological treatment for seizures, people with epilepsy (PwE) commonly experience impairments in quality of life (QoL). Given the limited access to psychosocial treatments for PwE, digital interventions could bridge treatment gaps and help improve QoL. The objective of this study was to examine the effectiveness of *emyna*, a fully automated digital intervention based on cognitive behavioral therapy (CBT) techniques, in improving health‐related QoL among PwE who reported impairments in QoL. A previous trial showed that *emyna* was effective in improving depressive symptoms among PwE with a comorbid depressive disorder, but its effects on QoL among PwE without comorbid depression remain unknown.

**Methods:**

A pragmatic randomized controlled trial was conducted with *N* = 438 PwE (mean age = 37.5, 70.3% women, physician‐verified diagnoses) who were assigned to the intervention group (*n* = 216), which used *emyna* alongside treatment as usual (TAU), or the control group (*n* = 222), which received TAU only. QoL and secondary outcomes such as general self‐efficacy, medication adherence, general distress, and epilepsy‐related work and social adjustment were assessed at baseline, 3 months, and 6 months. The primary outcome was QoL assessed with the Quality of Life in Epilepsy [QOLIE‐31] total score at 3 months post‐randomization.

**Results:**

Findings from the intent‐to‐treat analyses showed that after 3 months, participants in the intervention group experienced significant and clinically relevant improvements in health‐related QoL compared to the control group (baseline‐adjusted group difference = 4.5; 95% CI = [2.0, 6.9], *p* < 0.001; Cohen's *d* = 0.32). Effects on secondary outcomes did not reach statistical significance.

**Significance:**

This study extends previous research by demonstrating that *emyna* facilitates improvements in QoL in a diverse group of PwE treated in routine care settings. This CBT‐based digital intervention therefore presents a convenient and cost‐effective addition to healthcare providers' treatment repertoire.

**Plain Language Summary:**

In our study, we tested a digital program called *emyna*, which conveys cognitive behavioral therapy (CBT) techniques to help improve the quality of life for people living with epilepsy. We found that those who used emyna alongside their usual treatments felt better about their quality of life compared to those who did not use the program. Emyna offers a new, convenient way for people with epilepsy to manage their condition, which can be used alongside currently available treatments.

**Key points**

- The digital intervention *emyna* significantly improved quality of life (QoL) in people with epilepsy (*p* < 0.001, Cohen's *d* = 0.32) at 3 months, compared to control.
- 33.9% of participants in the emyna group achieved clinically meaningful improvements in QoL versus 21.3% in controls (NNT = 7.9).
- No significant effects on secondary outcomes; however, per‐protocol analyses showed improvement in self‐efficacy and general distress.
- High user satisfaction with *emyna*: 67.1% reported high to very high satisfaction, with a program recommendation rate of 6.9/10.
- These findings underscore *emyna*'s potential as a convenient, effective addition to holistic care for people with epilepsy.

## 1 INTRODUCTION

Epilepsy affects approximately 50 million people worldwide, making it one of the most common neurological disorders globally.^1^ People with epilepsy (PwE) not only face the manifestations of seizures, but also a spectrum of psychosocial sequelae, including the unpredictability surrounding seizures, the apprehension of experiencing seizures in public settings, restrictions on independent living, the weight of social stigmatization, and the distress that often accompanies living with a lifelong, chronic condition.^2^, ^3^, ^4^ These psychosocial complications pose significant burden on PwE^3^, ^5^ and may contribute to the development of psychiatric comorbidities, particularly affective disorders.^6^, ^7^

It is recognized that psychiatric comorbidities have a negative impact on quality of life (QoL) in epilepsy, even more so than factors directly related to the disorder, such as seizure frequency.^8^, ^9^, ^10^ In addition, up to 35% of adult PwE continue to experience seizures despite receiving antiseizure medication^11^ and are at high risk of persistent impairment in QoL.^8^, ^9^, ^12^ Even in PwE in whom remission is attainable through pharmacotherapy, QoL remains low when it comes at the cost of intolerable adverse events.^3^ Thus, there is a growing recognition of the necessity for a comprehensive treatment approach that addresses psychological and social factors as well.^2^, ^3^, ^5^, ^13^, ^14^, ^15^

In practice, however, the psychosocial complaints of PwE often go unnoticed and untreated.^3^, ^16^ As a result, PwE, particularly those with drug‐resistant epilepsy, continue to report a greater degree of unmet mental health care needs and lower QoL compared to the general population,^5^, ^17^, ^18^ despite the availability of effective psychological interventions such as cognitive behavioral therapy (CBT) and mindfulness‐based approaches.^19^, ^20^ Barriers to the provision of mental health care in epilepsy treatment settings include a lack of qualified psychotherapists, time constraints,^3^, ^13^ and extended waiting times for outpatient psychotherapy.^21^ Moreover, PwE may avoid mental health care due to a reluctance to acknowledge psychosocial concerns, fear of further stigmatization, and physical access difficulties.^17^, ^22^

Innovative therapeutic approaches, such as digital interventions, hold promise in overcoming these barriers, offering advantages such as accessibility, convenience, flexibility, affordability, and low clinician burden, particularly when interventions are fully automated.^23^ Patients themselves often express interest in exploring digital self‐management approaches^17^, ^24^ and perceive them as helpful when provided.^25^

Despite the potential benefits, few studies have explored digital interventions to enhance QoL in PwE. Previous research includes an RCT using mindfulness‐based cognitive therapy (MCBT) delivered through telephone or web‐based platforms, which showed promising but statistically non‐significant QoL improvements over 10 weeks.^26^ Another RCT evaluating a psychoeducational self‐management intervention did not show improved QoL compared to a waitlist control group after 12 weeks.^27^ Similarly, an RCT with a self‐guided digital intervention for depression (*deprexis*) did not enhance QoL in PwE, prompting the development of *emyna*, a self‐guided CBT‐based digital intervention tailored to the needs of PwE.^28^ Previous research has shown that, when used alongside TAU, *emyna* not only reduced depressive symptoms but also improved QoL in PwE with at least moderate depressive symptoms.^29^ The present RCT built upon these results and sought to assess *emyna*'s effectiveness in enhancing QoL for a broader range of PwE, including those without comorbid depression, within routine care settings.^30^ The primary hypothesis was that *emyna*, as an adjunct to TAU, would lead to greater QoL improvements after 3 months post‐randomization compared to TAU alone. Secondary hypotheses examined *emyna*'s effects on self‐efficacy, medication adherence, general distress, and social/work functioning after 3 and 6 months.

## 2 METHODS

### 2.1 Trial design

This study was designed as a parallel‐group, pragmatic RCT, assessing the effectiveness of the digital intervention *emyna* when used alongside treatment as usual (TAU). Patients in the intervention group received access to *emyna* directly following randomization for 6 months. The outcome time point of primary interest was 3 months post‐randomization. Additionally, online assessments were carried out 6 months after randomization. Following the 6‐month assessment, the TAU group was given the opportunity to access *eymna* (control group).

### 2.2 Trial setting and recruitment

We recruited participants using online ads, flyers, and health insurance provider promotions for an online trial. Interested individuals visited a dedicated study website for details and submitted their name and email. The study staff then contacted them via email for the baseline assessment. Recruitment took place from December 2021 to April 2022.

### 2.3 Standard protocol approvals, registrations, and patient consents

The trial was pre‐registered in a trial registry (DRKS.de: DRKS00025828) and approved by the Ethics Committee of the Charité – Universitätsmedizin Berlin (reference number: EA2/216/21). All study procedures followed ethical standards, including those from the 1964 Helsinki declaration and its amendments. Participants provided online consent.

### 2.4 Eligibility criteria

Eligibility criteria were (1) age of at least 18 years; (2) diagnosis of epilepsy (ICD‐10 code: G40.x, verified by physician's document); (3) prescription of antiseizure medication; (4) impairments in health‐related QoL (<67 on the Quality of Life in Epilepsy [QOLIE‐31] total score,^31^, ^32^ representing the lower two‐thirds of the questionnaire, which ranges from 0 to 100); and (5) consent to participation. No specific exclusion criteria were set, other than the need to have adequate German language proficiency and Internet access.

### 2.5 Randomization and blinding

We used a simple 1:1 randomization process with automatic sequence generation, maintaining concealment from study staff. Participants were not blinded to their group assignment.

### 2.6 Intervention

A comprehensive description of the structure and content of *emyna* has been published previously.^33^ In brief, *emyna* is an Internet‐based program designed specifically for PwE, serving as a self‐guided digital health application that requires no clinician support. *emyna* consists of five modules that provide information on epilepsy, its symptoms, psychosocial consequences, and introduces CBT exercises and techniques to integrate into daily life. The content presented to users is personalized based on their reported needs and interests. The core function of *emyna* is a “simulated dialog”, where users engage in a dynamic “conversation” with the program: Users are presented with brief text passages and select the response option that best resonates with them or matches their individual situation. *emyna* responds by providing a suitable next piece of information, resulting in a continuous dialog. In addition to the dialogues, *emyna* encourages users to complete simple homework tasks, offers audios, PDF worksheets, motivational messages delivered by e‐mail or SMS, and self‐monitoring questions. After the completion of our first *emyna* trial,^29^ which targeted only PwE with comorbid depression, some text revisions were made to optimize the program's suitability for PwE who may not be suffering from comorbid depressive symptoms.

### 2.7 Measures

Baseline data, as well as follow‐up data at 3 and 6 months, were collected using a secure and encrypted online survey service. Participants were sent e‐mail invitations to complete the assessments. For those who did not respond, up to three reminders were sent to encourage their participation.

#### 2.7.1 Primary outcome

The primary outcome was health‐related QoL in PwE, assessed with the QOLIE‐31 total score^32^ at 3 months post‐randomization.

#### 2.7.2 Secondary outcomes

Secondary outcomes included (1) general self‐efficacy, assessed with the Generalized Self‐Efficacy Scale (GSE)^34^; (2) medication adherence, assessed with the Rief Adherence Index (RAI)^35^; (3) general distress (depression, stress, and anxiety), as assessed by the Depression Anxiety Stress Scales (DASS)–21 items total score^36^; (4) epilepsy‐related social‐occupational impairment, measured by the Work and Social Adjustment Scale (WSAS).^37^

### 2.8 Sample size

An a priori power analysis was conducted with an estimated anticipated effect size of Cohen's *d* = 0.30, based on data from the previous *emnya*‐RCT conducted by our group.^29^ Considering an expected dropout rate of 20%, our goal was to randomize a total of *N* = 440 (2 × 220) participants, aiming for a statistical power of 0.80 with an alpha level of 0.05 (two‐tailed).

### 2.9 Statistical analysis

Analyses were conducted using *R*, version 4.3.0.

We performed intent‐to‐treat (ITT) analyses for all outcomes, which included all participants in the intervention and control groups, regardless of their usage of the intervention. Per‐protocol (PP) analyses, on the other hand, included only intervention group participants who registered to use *emyna*. To account for missing data at the primary endpoint of 3 months, we employed multiple imputation based on sociodemographic and clinical data (sex, age, family situation, employment, education, and psychotherapy utilization at baseline), following recommendations by Jakobsen et al.^38^ The imputation process was carried out using the *mice* and *bootImpute* packages in *R*.^39^, ^40^ Additionally, we conducted a conservative sensitivity analysis by employing the jump‐to‐reference (J2R) imputation method where missing values were imputed assuming that, following drop out, participants in the intervention group behave like those in the control group.^41^, ^42^

Since we examined two time points (baseline and 3 months), we employed analyses of covariance (ANCOVA), which are recommended for trials with pre‐ and post‐measurements.^43^ We assessed the effect of group (intervention vs. control) on the outcome variable at the 3‐month time point, while adjusting for baseline scores. The reported treatment effects represent mean group differences in the outcome variable at 3 months, adjusted for baseline scores, and are presented on the original scale alongside their corresponding 95% confidence intervals (CI). The statistical significance of these treatment effects was determined using *p*‐values obtained from the ANCOVA. Furthermore, we calculated between‐group effects (Cohen's *d*) by comparing the unadjusted mean values between the intervention and control groups at the 3‐month time point.^44^, ^45^ Similar analyses were performed for the 6‐month time point to assess the durability of effects.

We conducted a responder analysis for the primary endpoint, health‐related QoL assessed with the QOLIE‐31 total score after 3 months.^46^ We defined meaningful clinical improvement based on the minimally important clinical difference (MCID) previously reported for the QOLIE‐31.^47^ Specifically, we considered an individual improvement of at least 11.8 points in the QOLIE‐31 total score from baseline to the 3‐month time point as indicative of a response to treatment. The responder rates of the intervention and control groups were compared using a *χ* ^2^‐test.

Results were considered significant at a two‐sided 5% level.

## 3 RESULTS

### 3.1 Description of trial participants

Demographic and clinical characteristics are presented in Table 1. The majority of participants (70.3%) were women and had an average age of 37.5 years (SD = 13.9). Participants in both the intervention and control group exhibited comparable characteristics. The distribution of diagnostic subgroups at baseline is presented in Table S1. Relevant treatment characteristics over the course of the study are shown in Table 2.

**TABLE 1:** Subject demographics and clinical characteristics at baseline.

	Control	*Emyna*	Total
	*n* = 222	*n* = 216	*N* = 438
Age (in years)	37.7 (14.3)	37.3 (13.6)	37.5 (13.9)
Sex (% women)	72.1	68.5	70.3
Family situation (%)			
Unmarried	58.6	56.5	57.1
Married	33.3	32.9	33.1
Divorced/separated	8.2	8.8	8.5
Widowed	0	1.9	0.9
Employment (%)			
Full‐time	34.7	28.7	31.7
Part‐time	13.5	15.3	14.4
Marginal employment	5.0	4.6	4.8
Unemployed	29.3	35.6	32.4
Other employment	17.6	15.7	16.7
Education (%)			
No school‐leaving qualification	0.9	1.9	1.4
School‐leaving qualification (excl. higher education entrance qualification)	23.4	28.3	25.8
Higher education entrance qualification	20.3	16.7	18.5
Completed vocational training	25.2	24.1	24.7
Completed University studies	25.7	23.6	24.7
Other education	4.5	5.6	5.0
In psychotherapy (%)			
Current	22.1	27.3	24.7
Ever	57.2	56.5	56.8
Current antiseizure medication^a^ (%, multiple answers possible)			
Lamotrigine	52.8	50.5	51.6
Levetiracetam	42.1	43.7	42.9
Lacosamide	14.8	18.9	16.9
Valproic acid	12.0	11.3	11.6
Brivaracetam	8.3	10.4	9.4
Oxcarbazepine	6.5	5.9	6.2
Perampanel	5.6	6.3	5.9
Topiramate	6.0	4.5	5.3
Current anxiolytic medication^b^ (%)	1.7	0.7	1.3
Current antidepressive medication^c^ (%)	13.2	7.9	10.8
Other antiseizure treatment (%, multiple answers possible)			
Vagus nerve stimulation	1.4	0	0.7
Resective surgery	1.4	3.2	2.3
Deep brain stimulation	0	0	0
Ketogenic diet	1.8	1.9	1.8
Cannabis oil	6.3	3.7	5.0

*Note*: Values represent mean (SD) unless stated otherwise.

^a^ Only substances reported by >5% of patients in the total sample are shown.

^b^ Classified based on the Anatomical Therapeutic Chemical (ATC) Classification System (ATC code N05B).

^c^ Classified based on the Anatomical Therapeutic Chemical (ATC) Classification System (ATC code N06A).

**TABLE 2:** Relevant treatment characteristics over the course of the study.

	Control	*Emyna*	Statistical comparison
In psychotherapy (t1; current, %)	24.0	27.7	*χ* ^2^ = 0.64, *p* = 0.423
In psychotherapy (t2; current, %)	29.1	30.7	*χ* ^2^ = 0.10, *p* = 0.747
Change in antiseizure medication (baseline to t1; %, multiple answers possible)			
Reduction in dosage of existing medication^a^	23.1	16.0	*χ* ^2^ = 1.82, *p* = 0.178
Increase in dosage of existing medication^a^	24.6	35.0	*χ* ^2^ = 2.99, *p* = 0.084
Substance added^b^	10.8	12.3	*χ* ^2^ = 0.23, *p* = 0.629
Substance removed^b^	11.3	13.2	*χ* ^2^ = 0.26, *p* = 0.607
Change in antiseizure medication (t1 to t2; %, multiple answers possible)			
Reduction in dosage of existing medication^c^	6.7	14.8	*χ* ^2^ = 3.08, *p* = 0.079
Increase in dosage of existing medication^c^	11.1	13.6	*χ* ^2^ = 0.32, *p* = 0.572
Substance added^d^	6.0	12.2	*χ* ^2^ = 3.40, *p* = 0.065
Substance removed^d^	7.2	5.7	*χ* ^2^ = 0.23, *p* = 0.635
Other antiseizure treatment (t1; %, multiple answers possible)			
Vagus nerve stimulation	1.0	0	n/a
Resective surgery	2.0	3.1	*χ* ^2^ = 0.11, *p* = 0.744
Deep brain stimulation	0	0	n/a
Ketogenic diet	4.6	2.5	*χ* ^2^ = 0.55, *p* = 0.458
Cannabis oil	7.6	5.7	*χ* ^2^ = 0.27, *p* = 0.604
Other antiseizure treatment (t2; %, multiple answers possible)			
Vagus nerve stimulation	2.8	0.7	*χ* ^2^ = 0.95, *p* = 0.329
Resective surgery	1.7	2.8	*χ* ^2^ = 0.08, *p* = 0.772
Deep brain stimulation	0.6	0.7	*χ* ^2^ < .001, *p* = 1
Ketogenic diet	2.8	2.8	*χ* ^2^ < .001, *p* = 1
Cannabis oil	6.1	6.9	*χ* ^2^ = 0.004, *p* = 0.953
Experienced at least one seizure (%)			
From baseline to t1^e^	48.4 (1 seizure: 12.9, 2 seizures: 12.9, 3 seizures: 3.2, ≥4 seizures: 19.4)	64.5 (1 seizure: 25.8, 2 seizures: 12.9, 3 seizures: 9.7, ≥4 seizures: 16.1)	*χ* ^2^ = 1.64, *p* = 0.200
From t1 to t2^f^	51.5 (1 seizure: 9.6, 2 seizures: 9.6, 3 seizures: 5.6, ≥4 seizures: 26.9)	49.2 (1 seizure: 8.5, 2 seizures: 4.6, 3 seizures: 8.5, ≥4 seizures: 27.7)	*χ* ^2^ = 0.15, *p* = 0.698
Experienced change in nature and/or severity of seizures (%)			
From baseline to t1^e^	25.0	31.3	*χ* ^2^ = 0.08, *p* = 0.781
From t1 to t2^f^	20.4	22.0	*χ* ^2^ = 0.04, *p* = 0.844

^a^ Data on dosage for both t0 and t1 was available for *n* = 234 participants.

^b^ Data was available for *n* = 346 participants.

^c^ Data on dosage for both t1 and t2 was available for *n* = 223 participants.

^d^ Data was available for *n* = 289 participants.

^e^ Data on the number and nature of seizures from baseline to t1 was available for *n* = 62 participants.

^f^ Data on the number and nature of seizures from t1 to t2 was available for *n* = 299 participants.

### 3.2 Intervention delivery and participant retention

**FIGURE 1:**
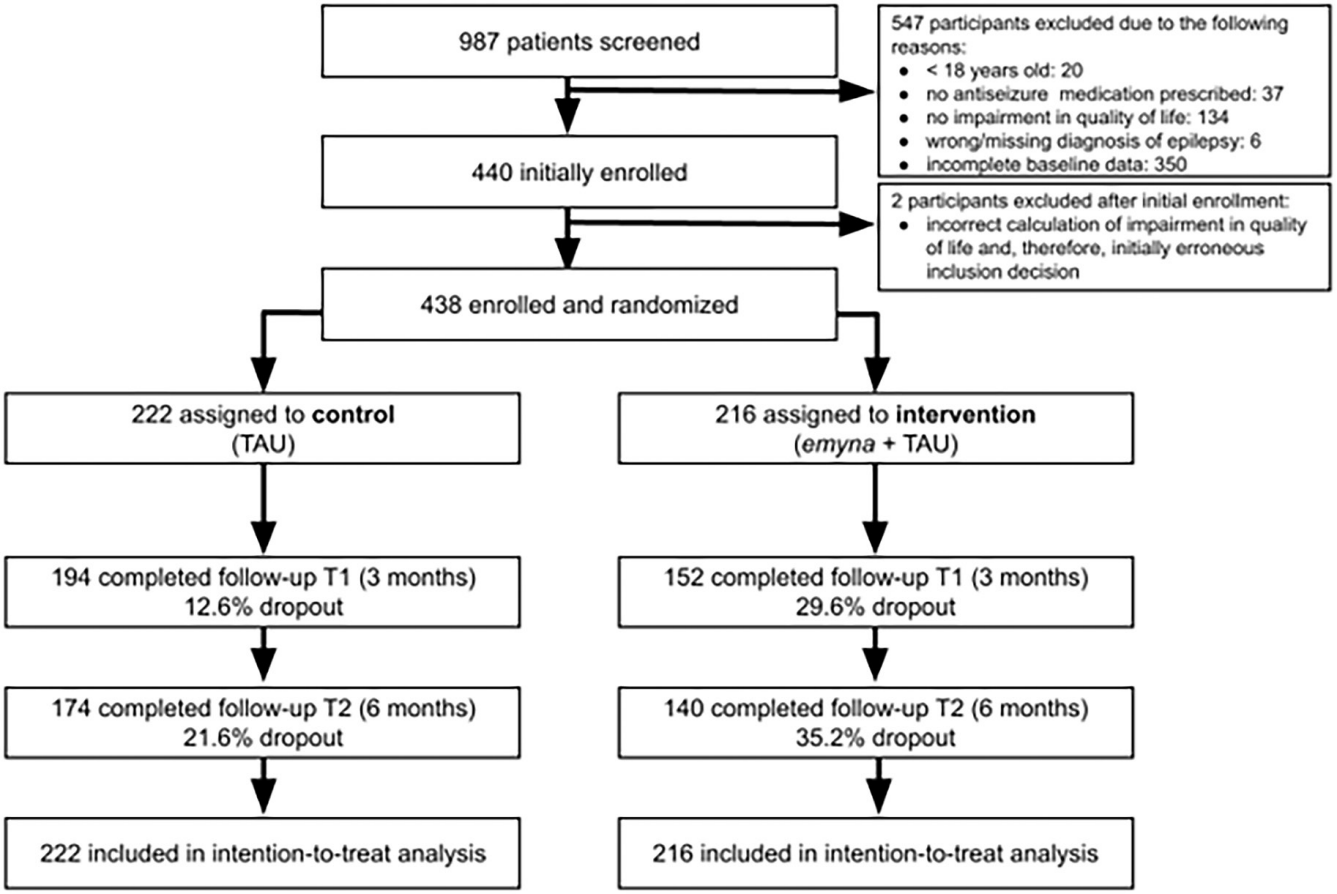
Participant flow through the study. TAU, Treatment as usual. T1: Follow‐up after 3 months; T2: Follow‐up after 6 months.

The participant flow is shown in Figure 1. Of the 987 individuals who started the screening questionnaire, 547 were excluded for various reasons (for a breakdown, see Figure 1). Additionally, two participants initially enrolled were not included in the statistical analyses due to an error in calculating the QOLIE‐31 total score which resulted in an incorrect inclusion decision for these participants. Among the 438 randomized participants, 346 (79.0%) completed primary outcome measures at 3 months, resulting in a 21.0% dropout rate. In the intervention group, 21 individuals (of 216 assigned) who did not sign up for *emyna* were excluded from the PP analysis.

### 3.3 Study outcomes

#### 3.3.1 Primary outcome: Health‐related QoL

**FIGURE 2:**
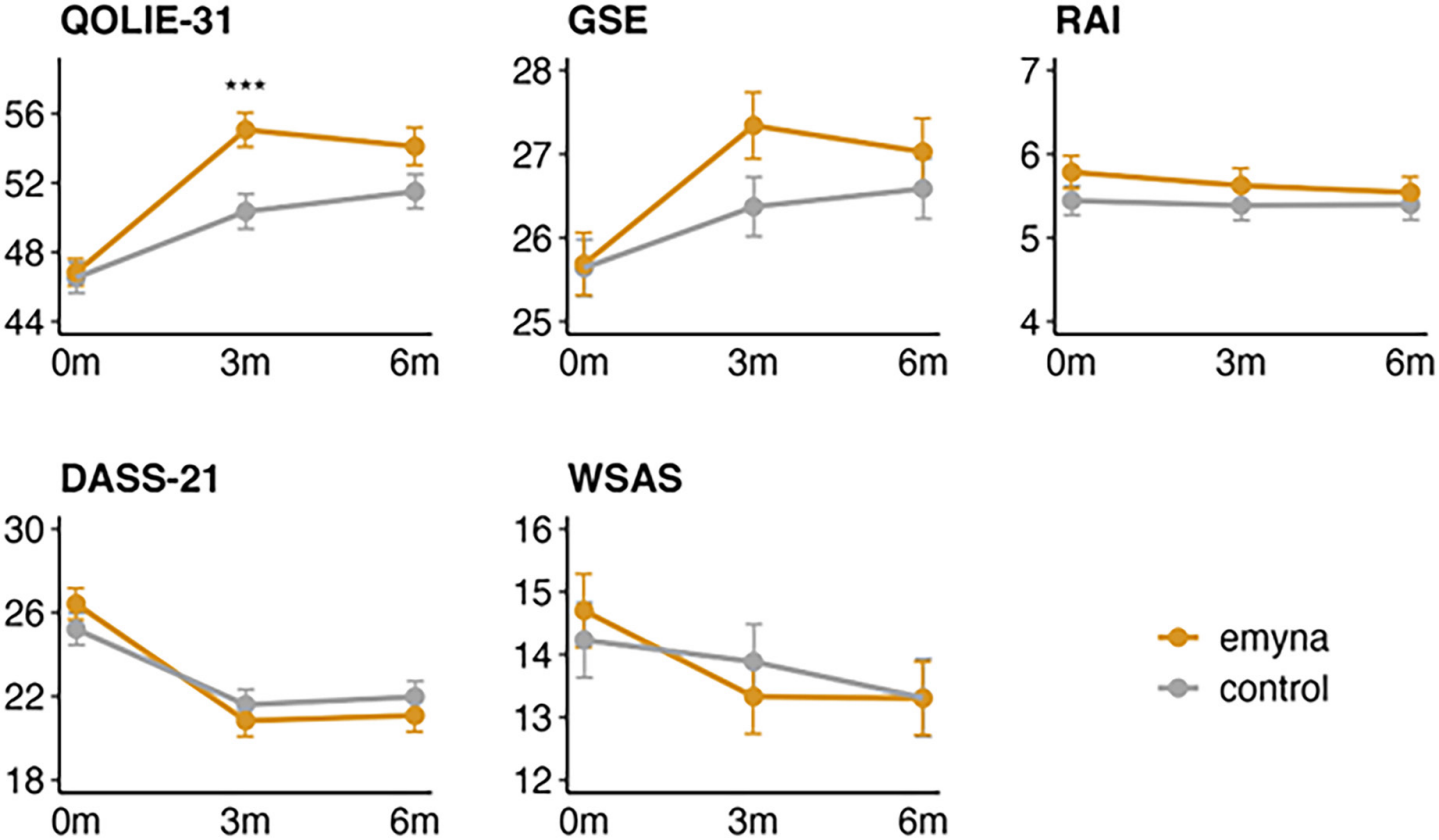
Symptom course on primary and secondary outcomes (means ± standard errors) using intent‐to‐treat analyses with multiple imputation for missing data. DASS‐21, Depression Anxiety Stress Scales–21 items; GSE, General Self‐Efficacy Scale; RAI, Rief Adherence Index; QOLIE‐31, Quality of Life in Epilepsy–31 items; WSAS, Work and Social Adjustment Scale. Plotted scores represent total scores. Asterisks indicate the level of statistical significance: ***p < 0.001. *p*‐values are derived from Analysis of Covariance (ANCOVA).

Results of the ITT analysis revealed that after 3 months of *emyna* use alongside TAU, the intervention group reported significantly higher health‐related QoL compared to the TAU‐only control group: the estimated baseline‐adjusted difference between the groups after 3 months was 4.5 points on the QOLIE‐31 total score (95% CI = [2.0, 6.9], *p* < 0.001; Cohen's *d* = 0.32; see also Table 3 and Figure 2). This effect was slightly larger in PP analyses (estimated baseline‐adjusted group difference on the QOLIE‐31 total score after 3 months = 5.0 points, 95% CI = [2.5, 7.6], *p* < 0.001; *d* = 0.34; see also Table S2 and Figure S1). As expected, the treatment effect was slightly smaller in the conservative J2R sensitivity analysis (estimated baseline‐adjusted group difference on the QOLIE‐31 total score after 3 months = 3.1, 95% CI = [1.3, 4.9], *p* < 0.001; *d* = 0.23; for details, see Table S3).

**TABLE 3:** Results from the intent‐to‐treat (ITT) analyses.

Time	Control (*n* = 222)			*Emyna* (*n* = 216)			ANCOVA		
	Mean	SD	Pre‐post Cohen's *d* (95% CI)	Mean	SD	Pre‐post Cohen's *d* (95% CI)	Treatment effect (95% CI)^a^	*p*‐Value	Between‐groups Cohen's *d* (95% CI)^b^
Primary endpoint: Quality of life (QOLIE‐31 total score)									
Baseline	46.5	13.2	–	46.8	11.6	–	–	–	–
3 months	50.4	15.0	0.35 (0.20, 0.50)	55.1	14.5	0.67 (0.49, 0.85)	4.5 (2.0, 6.9)	<.001	0.32 (0.11, 0.53)
6 months	51.5	14.7	0.43 (0.27, 0.58)	54.1	16.0	0.52 (0.35, 0.7)	2.4 (−0.4, 5.1)	0.089	0.17 (−0.04, 0.38)
Secondary endpoint: Self‐efficacy (GSE total score)									
Baseline	25.6	5.1	–	25.7	5.5	–	–	–	–
3 months	26.4	5.3	0.17 (0.04, 0.31)	27.3	5.8	0.33 (0.18, 0.48)	0.9 (−0.1, 1.9)	0.064	0.17 (−0.03, 0.38)
6 months	26.6	5.3	0.23 (0.08, 0.38)	27.0	5.9	0.28 (0.13, 0.43)	0.4 (−0.6, 1.4)	0.418	0.08 (−0.13, 0.29)
Secondary endpoint: Adherence (RAI total score)									
Baseline	5.4	2.6	–	5.8	2.9	–	–	–	–
3 months	5.4	2.7	0.07 (−0.01, 0.14)	5.6	3.0	0.08 (−0.02, 0.19)	0.1 (−0.5, 0.7)	0.777	0.08 (−0.13, 0.29)
6 months	5.4	2.7	0.06 (−0.01, 0.14)	5.5	2.8	0.1 (−0.01, 0.22)	0 (−0.5, 0.4)	0.839	0.05 (−0.14, 0.24)
Secondary endpoint: General distress (DASS‐21 total score)									
Baseline	25.2	11.2	–	26.4	10.9	–	–	–	–
3 months	21.6	10.8	0.39 (0.27, 0.52)	20.8	11.2	0.50 (0.34, 0.65)	−1.4 (−3.5, 0.6)	0.168	−0.07 (−0.29, 0.14)
6 months	22.0	11.0	0.33 (0.2, 0.47)	21.1	11.5	0.50 (0.34, 0.67)	−1.6 (−3.6, 0.3)	0.107	−0.08 (−0.29, 0.13
Secondary endpoint: Social and work‐related functioning (WSAS total score)									
Baseline	14.2	8.9	–	14.7	8.6	–	–	–	–
3 months	13.9	8.9	0.08 (−0.02, 0.18)	13.3	8.7	0.21 (0.05, 0.36)	−0.9 (−2.2, 0.4)	0.179	−0.06 (−0.27, 0.14)
6 months	13.3	9.2	0.15 (0.01, 0.29)	13.3	8.6	0.2 (0.04, 0.36)	−0.3 (−1.8, 1.1)	0.638	0 (−0.21, 0.21)

Abbreviations: ANCOVA, analysis of covariance; CI, confidence interval; DASS‐21, Depression Anxiety Stress Scales–21 items; GSE, General Self‐Efficacy Scale; RAI, Rief Adherence Index; SD, standard deviation; QOLIE‐31, Quality of Life in Epilepsy–31 items; WSAS, Work and Social Adjustment Scale.

^a^ Group difference on original scale 3 / 6 months after baseline, adjusted for baseline scores.

^b^ Based on unadjusted values; positive values show effects in favor of the intervention group.

Clinically meaningful improvements after 3 months were observed in 33.9% of the intervention group versus 21.3% in the control group (*χ* ^2^ = 8.70, *p* = 0.003), resulting in a Number Needed to Treat (NNT) of 7.9.^48^

#### 3.3.2 Secondary outcomes

After 3 months, the ITT analyses (see Table 3 and Figure 2) provided no clear evidence of a significant improvement in the intervention group compared to the control group for the secondary endpoints general self‐efficacy, medication adherence, general distress, and social and work‐related functioning.

PP analyses (see Table S2 and Figure S1) showed significant effects of *emyna* on general self‐efficacy (estimated baseline‐adjusted group difference on the GSE after 3 months = 1.1 points, 95% CI = [0.1, 2.1], *p* = 0.028; *d* = 0.21) and general distress (estimated baseline‐adjusted group difference on the DASS‐21 total score after 3 months = −2.1 points, 95% CI = [−4.1, 0], *p* = 0.047; *d* = 0.14). PP results for the other secondary endpoints were comparable with the ITT analysis. Results from the conservative J2R suggested overall smaller estimates of treatment effects than did ITT results, as can be expected (see Table S3).

No adverse events or adverse device effects were observed. As an additional safety analysis, we calculated the percentage of participants who showed a decline in QoL from baseline to 3 months. This analysis was based on participants with complete observations only (*n* = 346). 25.7% of participants in the intervention group compared to 33.0% of participants in the control group reported a lower QoL at 3 months compared to baseline (*χ* ^2^ = 2.19, *p* = 0.139).

#### 3.3.3 Stability of effects after 6 months

After 6 months, patients in the intervention group reported descriptively higher health‐related QoL than patients in the control group, but the group difference was not statistically significant in the ITT analysis (baseline‐adjusted group difference in the QOLIE‐31 total score = 2.4 points, 95% CI = [−0.4, 5.1], *p* = 0.089; *d* = 0.17; see also Table 3 and Figure 2). However, the group difference reached significance in the PP analysis (baseline‐adjusted group difference in the QOLIE‐31 total score = 3.0 points, 95% CI = [0.1, 5.8], *p* = 0.044; *d* = 0.20; see also Table S2).

The ITT analyses (see Table 3 and Figure 2) provided no evidence of an intervention effect of *emyna* on the secondary outcomes general self‐efficacy, medication adherence, general distress, and social and work‐related functioning. The PP analyses (see also Table S2 and Figure S1) yielded similar results for most secondary outcomes, except for general distress, which showed a significant difference between the groups (estimated baseline‐adjusted group difference on the DASS‐21 total score after 6 months = −2.0 points, 95% CI = [−4.0, −0.1], *p* = 0.042; *d* = 0.13). As expected, results from the conservative J2R again suggested smaller estimates of treatment effects (see Table S3).

#### 3.3.4 User satisfaction and engagement with *emyna*

After 3 months of accessing *emyna*, participants in the intervention group were asked to rate their likelihood of recommending the program to a friend or colleague to assess user satisfaction.^49^ Ratings were recorded on an 11‐point Numerical Rating Scale (NRS), ranging from 0 (‘I definitely do not recommend the program’) to 10 (‘I definitely recommend the program’). Out of the *n* = 149 respondents, 12.1% expressed low satisfaction (NRS = 0–3), 20.8% indicated moderate satisfaction (NRS = 4–6), and 67.1% reported high to very high satisfaction (NRS = 7–10). These results indicate overall positive user satisfaction. Notably, *emyna* received an average rating of 6.9 (SD = 2.6), suggesting it was more often recommended than not. On average, participants used *emyna* on average over a span of 37 separate days within the 180‐day period during which program access was possible.

## 4 DISCUSSION

### 4.1 Summary of findings

This RCT demonstrated that the digital health application *emyna*, when used alongside TAU, significantly improved health‐related QoL after 3 months. Specifically, 33.9% of intervention group participants achieved a clinically significant enhancement in QoL compared to 21.3% in the control group, corresponding to an NNT of 7.9. Significant effects of *emyna* on the secondary outcomes general self‐efficacy and distress were shown in the PP analyses for up to 6 months, which included participants who had at least registered to access the digital intervention. The improvements in QoL tended to be sustained over a 6‐month period and were confirmed in a conservative sensitivity analysis.

These findings are encouraging, especially when compared to other interventions targeting QoL in epilepsy. Meta‐analytic evidence shows that psychological interventions for PwE delivered by clinicians have an average treatment effect of 5.2 points on health‐related QoL, as measured by the QOLIE‐31. Participants who used the digital intervention *emyna* alongside their usual treatment for 3 months reported QOLIE‐31 scores that improved by 8.3 points, on average, over the study period and, at the primary endpoint of 3 months, were 4.5 points higher compared to the control group. Thus, *emyna* facilitated improvements comparable to clinician‐led interventions.^23^ Building upon a previous RCT that only included PwE with comorbid depressive symptoms,^29^ the present study demonstrated the effectiveness of *emyna* in a more diverse group of patients, including those without comorbid depression.

The lack of significant results on secondary outcomes is somewhat surprising, although improvements in self‐efficacy and general distress were observed among those who registered for the intervention. Several factors may explain this. First, the concept of general self‐efficacy was assessed as a trait‐like personality characteristic, using the GSE with items such as “I can always manage to solve difficult problems if I try hard enough”. Changing trait‐like personality characteristics may pose challenges for any behavioral intervention. Our findings also align with previous research that reported no effect of a nurse‐led self‐management group intervention on self‐efficacy in PwE.^50^ Second, floor effects were observed in terms of medication adherence: At baseline, a significant proportion of participants demonstrated high adherence to the prescribed medication, leaving limited scope for improvement through the use of *emyna*. Third, the measure we used to assess epilepsy‐related work and social adjustment may not be sensitive to change because it specifically inquires about the extent of social‐occupational impairment due to epilepsy, which is a chronic disease. By contrast, our previous *emyna* trial had inquired about social‐occupational impairment due to depressive symptoms, which can fluctuate more than a chronic disease.^29^ Future research could disentangle intervention effects on social‐occupational impairments that are due to either fluctuating symptoms or due to epilepsy itself.

Finally, the smaller effects on general distress, compared to the previous *emyna* trial in PwE with comorbid depression,^29^ may be due to our use of a cut‐off in the QOLIE‐31 total score as an inclusion criterion. The QOLIE‐31 total score assigns relatively more weight to cognitive impairment (e.g., memory and concentration) compared to other domains of QoL. Consequently, the sample preferentially included PwE with relatively pronounced cognitive impairments, which might have made it particularly difficult for a CBT‐based, self‐guided intervention like *emyna* to facilitate improvements for several reasons: Firstly, this may have created a mismatch between the needs of PwE with cognitive difficulties and the structure of *emyna* as a primarily text‐based online program. Second, those with greater cognitive impairment may struggle with techniques requiring intact cognitive abilities (e.g., concentration, attention), such as mindfulness exercises. Efforts to improve the suitability of *emyna* for patients with cognitive impairments may involve integrating multimedia elements, such as simplified visual aids, and interactive features like prompts for cognitive exercises.

These findings add to the literature on the potential utility of CBT‐based interventions in epilepsy. Reviews of extant literature suggest that CBT may be considered as a low‐risk adjunct to standard epilepsy treatments, as some such interventions have documented their potential in terms of improving quality of life.^51^ Advantages such as the non‐invasive nature of these treatments, their potential to engage patients, and their low cost must be weighed against certain limitations, however, such as the limited availability of CBT for many patients, access barriers, and limited research support for most CBT‐based, epilepsy‐specific treatment approaches.^51^ In this study, only around 30% of participants were in current psychotherapy, confirming that the majority of patients have limited access to professional psychosocial support, consistent with the notion that there is a general unmet need for psychological treatment in many PwE.^13^, ^18^

### 4.2 Strengths and limitations

The present RCT exhibited several strengths. First, the trial adopted a pragmatic design that mirrored real‐world clinical practice. This design resulted in a limited set of inclusion and exclusion criteria, representing the heterogeneity of routine care conditions and maximizing external validity and generalizability. Second, the trial demonstrated adequate statistical power, ensuring that the sample size was sufficiently large to detect meaningful differences between the treatment groups. Third, the trial incorporated follow‐up assessments at two time points, extending up to 6 months post‐randomization.

We also acknowledge several limitations. First, the 21% dropout rate could introduce bias and limit generalizability. High attrition rates are common in online interventions for chronic health conditions such as epilepsy,^52^, ^53^ with a reported average dropout rate of 40% in RCTs testing app‐based interventions.^52^ Moreover, present dropout rates were comparable to those observed in the previous RCT assessing *emyna*'s effectiveness.^29^ Also, similar to the previous RCT, the intervention group had a higher dropout rate than the control group. This might be because participants in the intervention group discontinued study participation once they felt *emyna* provided sufficient benefit, aligning with the ‘good enough’ effect documented in psychotherapy^54^ and in digital interventions research.^55^ In contrast, the control group showed greater adherence to follow‐up assessments, possibly motivated by the prospect of being able to access *emyna* later. Understanding the reasons behind premature dropout could offer insights for improving digital interventions. Previous research suggests that variables such as age, gender, education, expectations, perceived participation burden, comorbid anxiety, preference for in‐person treatments, motivation, time constraints, and personalization of the intervention might all predict adherence.^53^, ^56^, ^57^

Second, our sample included a larger proportion of women compared to the typical epidemiological distribution observed in Germany.^58^ This aligns with the documented higher likelihood of women to seek psychosocial support.^59^, ^60^, ^61^ Specifically, data from Germany indicate that women are nearly twice as likely as men to utilize outpatient psychotherapy.^59^ This tendency extends to the digital realm, with up to 70% of all prescriptions for digital health applications in Germany going to women.^62^, ^63^

Third, the use of a TAU control group merits consideration. TAU control groups mirror clinical routine care and are valuable for assessing general intervention effectiveness, as noted by an NIH expert panel.^30^ Conversely, active control groups, like non‐specific sham interventions, might yield more nuanced insights into the causal mechanisms explaining the effects of *emyna*. However, designing sham or placebo interventions for psychosocial interventions is conceptually challenging as it necessitates the establishment of intervention credibility without including any active therapeutic elements.^64^, ^65^, ^66^

A fourth limitation is that this was not a developer‐independent trial; that is, several authors and research personnel are employed by the company that developed and owns the digital intervention examined in this study. Industry‐sponsored research generally tends to favor the product being investigated,^67^, ^68^ and investigator allegiance can introduce biases even in psychotherapy research, regardless of whether the study is funded by industry or the intervention developers.^69^ In the present study, several precautions were taken to prevent such bias from occurring. Firstly, the Principal Investigator (MH) and another coauthor (KB) are independent established researchers in the field of epilepsy without financial or other ties to the developers. Secondly, the trial was pre‐registered at a trial registry before participant recruitment commenced; and thirdly, the study protocol was reviewed and approved by an independent ethics committee. We also note in this context that a meta‐analysis of a similar digital intervention developed by our group has shown no significant difference in effect sizes reported in developer‐independent trials compared to those that included the developers.^70^ Therefore, we have confidence in the accuracy of the results reported in this trial, and we concur with others that scientific progress ultimately requires independent replication in methodologically sound studies.^71^

A final potential limitation is that this study did not examine whether personal guidance or supportive contacts might have enhanced the intervention's effectiveness, as some studies have suggested.^72^, ^73^ However, this potential advantage must be weighed against the greater personnel and resource requirements compared to fully automated online interventions like *emyna*. Moreover, prior work^70^, ^74^ has shown that the addition of personal support did not always enhance the effectiveness of similar digital interventions. Future research could examine whether adding personal support to *emyna* could improve the program's effectiveness on QoL and other outcomes.

In conclusion, the present study provides evidence that *emyna*, a fully automated digital intervention, is effective in improving QoL in a broad population of PwE at levels comparable to clinician‐led interventions. Considering its safety, effectiveness, accessibility, and potential for cost savings, *emyna* could serve as a meaningful addition to the treatment repertoire of epilepsy healthcare providers.

## AUTHOR CONTRIBUTIONS

B.M.: Conceptualization, Project Administration, Formal Analysis, Writing – Original Draft Preparation, Writing – Review & Editing. L.T.B.: Formal analysis, Visualization, Writing – Original Draft Preparation, Writing – Review & Editing. K.B., M.H.: Conceptualization, Writing – Review & Editing.

## FUNDING INFORMATION

This research was funded by GAIA, Hamburg, Germany, a research‐focused small‐to‐medium enterprise that specializes in e‐health interventions and regularly participates in publicly funded research. There was no external funding.

## CONFLICT OF INTEREST STATEMENT

B.M. and L.T.B. are affiliated with GAIA, the company that funded this trial and that developed, owns, and operates the digital intervention evaluated in it. B.M. is employed full‐time as Research Director, L.T.B. is employed full‐time as Senior Researcher. None of the authors who are not employed by GAIA (K.B., M.H.) has received any remuneration from GAIA. M.H. has received speaker honoraria and/or consultancy fees from Angelini, Bial, Desitin, Eisai, Jazz Pharma and UCB within the past 3 years. K.B. declares that she has no competing interests. We confirm that we have read the Journal's position on issues involved in ethical publication and affirm that this report is consistent with those guidelines.

## ETHICS APPROVAL STATEMENT

This trial was approved by the Ethics Committee of the Charité – Universitätsmedizin Berlin (reference number: EA2/216/21). All study procedures followed ethical standards, including those from the 1964 Helsinki declaration and its amendments.

## PATIENT CONSENT STATEMENT

All participants provided online consent.

## CLINICAL TRIAL REGISTRATION

The trial was pre‐registered on 08 December 2021 at the German Clinical Trials Registry (DRKS.de: DRKS00025828).

## Supporting information


Appendix S1.

## Data Availability

The data that support the findings of this study are available from the corresponding author upon reasonable request.
