# Supplementary material for: Enhancing quality of life in epilepsy with a digital intervention (emyna): Results of the ELAINE randomized controlled trial
Source: Epilepsia Open. 2024 Aug 21;9(5):1758–71. doi: 10.1002/epi4.13014 (PMC11450619; doi:10.1002/epi4.13014)
Supplement: Supplementary file 1 — Appendix S1. [file EPI4-9-1758-s001.docx]

**Supplementary material for**

**“Enhancing quality of life in epilepsy with a digital intervention (*emyna*): results of the ELAINE randomized controlled trial”**

Björn Meyer, Linda T. Betz, Katja Brückner, Martin Holtkamp


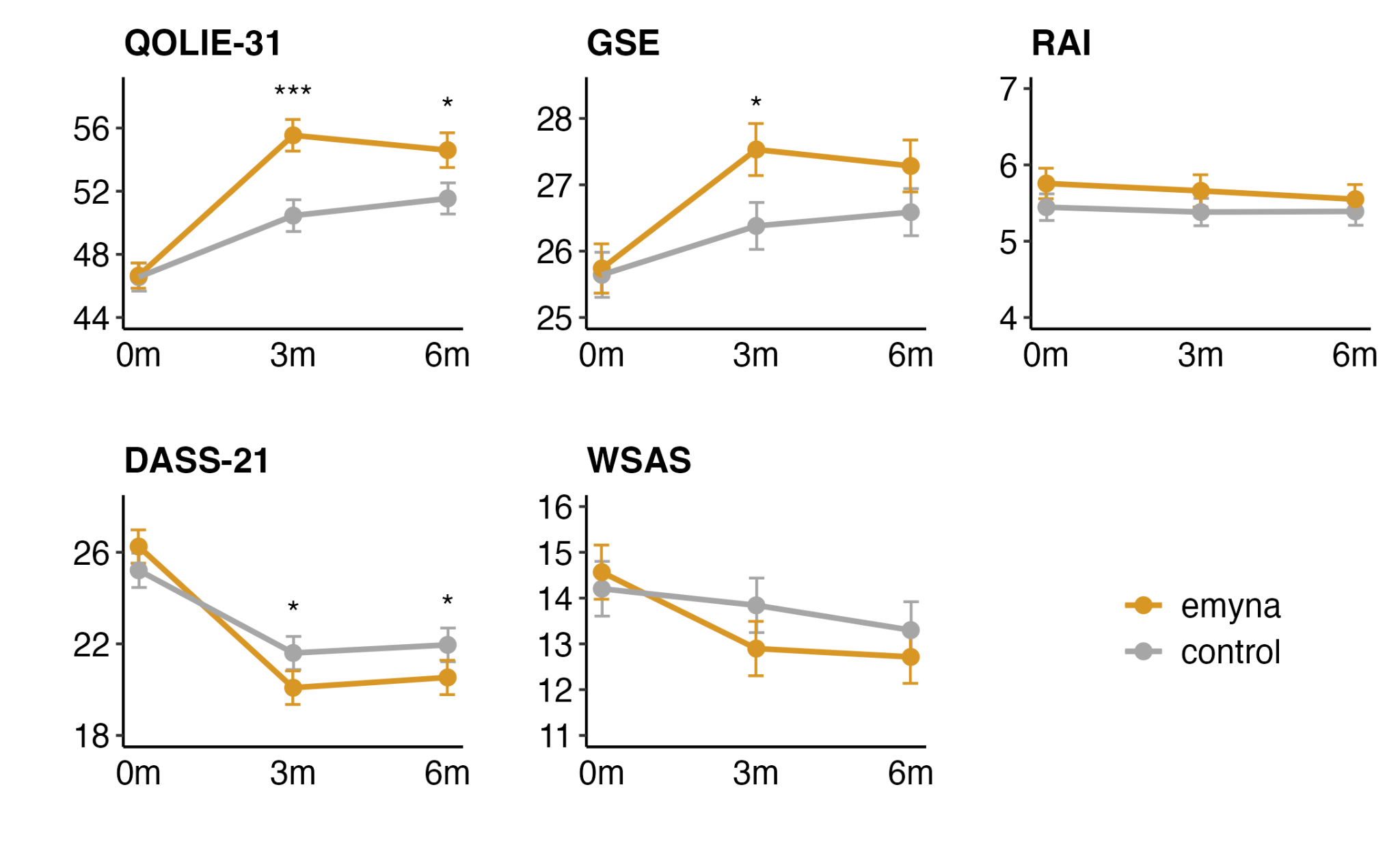


Supplementary Figure 1 *|* Symptom course on primary and secondary outcomes (means ± standard errors) using per protocol (PP) analyses with multiple imputation for missing data. DASS-21: Depression Anxiety Stress Scales–21 items; GSE: General Self-Efficacy Scale; RAI: Rief Adherence Index; QOLIE-31: Quality of Life in Epilepsy–31 items; WSAS: Work and Social Adjustment Scale. Plotted scores represent total scores. * *p* < 0.05, *** *p* < 0.001. *p*-values are derived from Analysis of Covariance (ANCOVA).

Supplementary Table 1 | Overview of the diagnostic subgroups in the study.

|  | **Control** | ***emyna*** | **Total** |
| --- | --- | --- | --- |
| **Diagnostic subgroup^a^**  **(ICD-10 code, %)** |  |  |  |
| G40.0 | 9.4 | 14.3 | 11.5 |
| G40.1 | 8.2 | 3.0 | 5.9 |
| G40.2 | 14.6 | 19.5 | 16.8 |
| G40.3 | 17.5 | 19.5 | 18.4 |
| G40.4 | 5.8 | 2.3 | 4.3 |
| G40.5 | 1.8 | 0 | 1.0 |
| G40.6 | 17.0 | 15.8 | 16.4 |
| G40.7 | 0.6 | 2.3 | 1.3 |
| G40.8 | 3.5 | 4.5 | 3.9 |
| G40.9 | 9.4 | 10.5 | 9.9 |
| G40  (epilepsy, not further specified) | 12.3 | 8.3 | 10.5 |

**^a^**  Data on diagnostic subgroups (ICD-10 codes) were available for n = 304 participants.

Supplementary Table 2 | Results from the per protocol (PP) analyses.

| **Time** | **Control**  **(n = 222)** | | | ***emyna***  **(n = 195)** | | | **ANCOVA** | |  |
| --- | --- | --- | --- | --- | --- | --- | --- | --- | --- |
|  | mean | SD | pre-post Cohen’s *d*  (95% CI) | mean | SD | pre-post  Cohen’s *d*  (95% CI) | Treatment effect  (95% CI)^a^ | *p*-value | between-groups  Cohen's *d*  (95% CI)^b^ |
| **Primary endpoint: Quality of life (QOLIE-31 total score)** | | | | | | | | | |
| Baseline | 46.6 | 13.2 | - | 46.7 | 11.7 | - | - | - | - |
| 3 months | 50.5 | 15.0 | 0.36  (0.21, 0.50) | 55.5 | 14.7 | 0.73  (0.54, 0.92) | 5.0  (2.5, 7.6) | <. 001 | 0.34  (0.13, 0.56) |
| 6 months | 51.5 | 14.7 | 0.43  (0.27, 0.59) | 54.6 | 16.2 | 0.58  (0.4, 0.76) | 3.0  (0.1, 5.8) | 0.044 | 0.20  (-0.02, 0.42) |
| **Secondary endpoint: Self-efficacy (GSE total score)** | | | | | | | | |  |
| Baseline | 25.6 | 5.1 | - | 25.7 | 5.5 | - | - | - | - |
| 3 months | 26.4 | 5.3 | 0.17  (0.03, 0.31) | 27.5 | 5.8 | 0.35  (0.2, 0.51) | 1.1  (0.1, 2.1) | 0.028 | 0.21  (0, 0.42) |
| 6 months | 26.6 | 5.3 | 0.23  (0.09, 0.38) | 27.3 | 5.7 | 0.32  (0.17, 0.48) | 0.6  (-0.3, 1.6) | 0.191 | 0.13  (-0.08, 0.34) |
| **Secondary endpoint: Adherence (RAI total score)** | | | | | | | | | |
| Baseline | 5.4 | 2.6 | - | 5.8 | 2.9 | - | - | - | - |
| 3 months | 5.4 | 2.7 | 0.07  (-0.01, 0.14) | 5.7 | 3.1 | 0.08  (-0.01, 0.17) | 0.1  (-0.5, 0.7) | 0.649 | 0.09  (-0.12, 0.31) |
| 6 months | 5.4 | 2.7 | 0.06  (-0.01, 0.14) | 5.6 | 2.8 | 0.09  (-0.01, 0.2) | 0  (-0.5, 0.4) | 0.923 | 0.06  (-0.14 |
| **Secondary endpoint: General distress (DASS-21 total score)** | | | | | | | | | |
| Baseline | 25.2 | 11.2 | - | 26.3 | 10.7 | - | - | - | - |
| 3 months | 21.6 | 10.8 | 0.40  (0.26, 0.53) | 20.1 | 10.8 | 0.54  (0.38, 0.71) | -2.1  (-4.1, 0) | 0.047 | 0.14  (-0.08, 0.36) |
| 6 months | 22.0 | 11.0 | 0.34  (0.2, 0.48) | 20.5 | 11.0 | 0.55  (0.38, 0.72) | -2.0  (-4, -0.1) | 0.042 | 0.13  (-0.08, 0.34) |
| **Secondary endpoint: Social and work-related functioning (WSAS total score)** | | | | | | | | | |
| Baseline | 14.2 | 8.9 | - | 14.6 | 8.7 | - | - | - | - |
| 3 months | 13.8 | 8.9 | 0.08  (-0.02, 0.18) | 12.9 | 8.7 | 0.25  (0.08, 0.42) | -1.2  (-2.6, 0.2) | 0.086 | 0.11  (-0.11, 0.32) |
| 6 months | 13.3 | 9.2 | 0.15  (0.01, 0.29) | 12.7 | 8.5 | 0.26  (0.08, 0.44) | -0.8  (-2.2, 0.6) | 0.266 | -0.07  (-0.14, 0.27) |

^a^ Group difference on original scale 3 / 6 months after baseline, adjusted for baseline scores.

^b^ based on unajdusted values; positive values show effects in favor of the intervention group.

*Abbreviations*: ANCOVA: analysis of covariance; CI: confidence interval; DASS-21: Depression Anxiety Stress Scales–21 items; GSE: General Self-Efficacy Scale; RAI: Rief Adherence Index; SD: standard deviation; QOLIE-31: Quality of Life in Epilepsy–31 items; WSAS: Work and Social Adjustment Scale.

Supplementary Table 3 | Results from the jump-to-reference (J2R) sensitivity analyses.

| **Time** | **Control**  **(n = 222)** | | | ***emyna***  **(n = 216)** | | | **ANCOVA** | |  |
| --- | --- | --- | --- | --- | --- | --- | --- | --- | --- |
|  | mean | SD | pre-post Cohen’s *d*  (95% CI) | mean | SD | pre-post  Cohen’s *d*  (95% CI) | Treatment effect  (95% CI)^a^ | *p*-value | between-groups  Cohen's *d*  (95% CI)^b^ |
| **Primary endpoint: Quality of life (QOLIE-31 total score)** | | | | | | | | | |
| Baseline | 46.6 | 13.2 | - | 46.9 | 11.6 | - | - | - | - |
| 3 months | 50.4 | 14.9 | 0.36  (0.20, 0.51) | 53.7 | 14.5 | 0.56  (0.43, 0.68) | 3.1  (1.3, 4.9) | <. 001 | 0.23  (0.05, 0.40) |
| 6 months | 51.7 | 14.4 | 0.45  (0.28, 0.62) | 53.0 | 15.9 | 0.46  (0.33, 0.59) | 1.1  (-0.7, 2.9) | 0.231 | 0.09 (-0.08, 0.26) |
| **Secondary endpoint: Self-efficacy (GSE total score)** | | | | | | | | |  |
| Baseline | 25.6 | 5.1 | - | 25.7 | 5.5 | - | - | - | - |
| 3 months | 26.4 | 5.2 | 0.18  (0.03, 0.32) | 27.1 | 5.8 | 0.27  (0.16, 0.39) | 0.6  (-0.1, 1.3) | 0.073 | 0.12  (-0.05, 0.29) |
| 6 months | 26.6 | 5.3 | 0.24  (0.08, 0.4) | 27.0 | 5.9 | 0.26  (0.14, 0.38) | 0.3  (-0.3, 0.9) | 0.356 | 0.06  (-0.11, 0.23) |
| **Secondary endpoint: Adherence (RAI total score)** | | | | | | | | | |
| Baseline | 5.4 | 2.6 | - | 5.8 | 2.9 | - | - | - | - |
| 3 months | 5.4 | 2.6 | 0.06  (-0.02, 0.15) | 5.6 | 2.9 | 0.07  (-0.02, 0.16) | 0.1  (-0.4, 0.5) | 0.805 | 0.08  (-0.09, 0.25) |
| 6 months | 5.4 | 2.8 | 0.06  (-0.01, 0.14) | 5.6 | 2.8 | 0.07  (-0.02, 0.16) | 0  (-0.3, 0.3) | 0.961 | 0.08  (-0.08, 0.24) |
| **Secondary endpoint: General distress (DASS-21 total score)** | | | | | | | | | |
| Baseline | 25.2 | 11.2 | - | 26.4 | 10.9 | - | - | - | - |
| 3 months | 21.5 | 10.8 | 0.40  (0.28, 0.53) | 21.3 | 11.2 | 0.46  (0.34, 0.58) | -0.9  (-2.3, 0.5) | 0.209 | -0.02  (-0.19, 0.15) |
| 6 months | 21.8 | 11.0 | 0.37  (0.23, 0.5) | 22.0 | 11.6 | 0.41  (0.3, 0.53) | -0.5  (-1.7, 0.8) | 0.465 | 0.02  (-0.14, 0.18) |
| **Secondary endpoint: Social and work-related functioning (WSAS total score)** | | | | | | | | | |
| Baseline | 14.2 | 8.9 | - | 14.7 | 8.6 | - | - | - | - |
| 3 months | 13.9 | 8.9 | 0.08  (-0.03, 0.18) | 13.5 | 8.8 | 0.17  (0.05, 0.29) | -0.6  (-1.6, 0.3) | 0.178 | -0.03  (-0.21, 0.14) |
| 6 months | 13.1 | 9.2 | 0.19  (0.04, 0.33) | 13.4 | 8.8 | 0.18  (0.06, 0.31) | 0  (-0.9, 0.9) | 0.979 | 0.04  (-0.13, 0.21) |

^a^ Group difference on original scale 3 / 6 months after baseline, adjusted for baseline scores.

^b^ based on unajdusted values; positive values show effects in favor of the intervention group.

*Abbreviations*: DASS-21: Depression Anxiety Stress Scales–21 items; GSE: General Self-Efficacy Scale; RAI: Rief Adherence Index; QOLIE-31: Quality of Life in Epilepsy–31 items; WSAS: Work and Social Adjustment Scale.
